# 

**Published:** 1909-08-15

**Authors:** 


### ANNOUNCEMENTS.

SECOND MICHIGAN DISTRICT DENTAL SOCIETY. The annual election resulted as follows: Dr. Don M. Graham, President; Dr. R. M. Muir, Vice President; Dr. J. A. Walker, Secretary; Dr. T. R. Buttrick, Treasurer; Dr. W. A. Giffen, Censor (3 yrs.) in place of Dr. Hogarth; Dr. M. V. Hopkins, Censor, 2 years; Dr. G. B. Watkins, Censor, 1 year.

DENTAL METALLURGY. Lea & Febiger of Philadelphia, have just printed and have ready for distribution the sixth edition of "A Manual of Dental Metallurgy," by Essig & Koenig.

It is thoroughly revised in all its parts and up to date. It has also been considerably enlarged, much new matter being added as well as retaining the valuable features of the fifth edition. A detailed study of the qualatative analysis of metals of interest to dentists has been incorporated. Also a series of experiments relative to the physical properties of metals has been arranged for the use of students in dental colleges or individuals wishing to pursue a practical study of metallurgy.

This sixth edition seems to be a very complete laboratory guide, a practical working manual and a book of reference and instruction.

---

GORGAS DENTAL MEDICINE. There has just come from the press of P. Blakiston's Son & Co., the eighth edition of Gorgas Dental Medicine. This is the oldest and best known text book on this subject and has been more extensively used than all other books of its kind. The form of treating the matter has not been materially changed in the edition. A very handy and useful table of doses has been added from Gould's Dictionary and many new drugs and dental remidies are added as well as a large number of remedies that have appeared in our current literature. The one fault we have always found with this work for class purposes is its lack of classifying the remedies into groups with kindred pharmachological action. The plan of listing each one in its alphabetical order is convenient for the practitioner, who only consults such books for specific information. The listing of formulae accredited to their respective authors is valuable as it enables one to quickly refer to them. The book is well made as are all the books from this house, and because it puts in concise form the salient facts as to modern drugs and remedies it should have a large sale. The price is four dollars which is insignificant when compared with the value.

---

Pyorrhoea Alveolaris. A committee to investigate the present status of this disease as viewed by the profession was appointed last year by the Louisiana State Society, and has just made a report, a copy of which we have received. It is somewhat fragmentary as it is catagorical in form, and while furnishing a variety of personal opinions, it gives no substantial scientific basis for maturing judgment. We hope it may serve to stimulate definite research.

---

Missouri State dental Association. At the forty-fourth meeting of the Missouri State Dental Association held in Kansas City, May 26th, 27th and 28th, 1909, a resolution was adopted to reorganize the state association and redistrict the state and a strong committee was appointed for that purpose, to report at the next meeting. The following officers were elected: President, R. E. Darby, Springfield; First Vice-President, O. J. Fruth, St. Louis; Recording Secretary, J. F. Wallace, Canton; Corresponding Secretary, F. W. Patterson, Tipton; Treasurer, J. T. Fry, Moberly. The meeting next year will be held in St. Louis.

F. W. Patterson, *Cor. Sec.*
Tipton, Mo.

